# Shifting focus: the need for early intervention and safer alternatives in autism spectrum disorder treatment

**DOI:** 10.47626/2237-6089-2024-0953

**Published:** 2025-01-07

**Authors:** Muhammad Hunain Raza, Muhammad Eeman Bhutta, Muhammad Hammad Siddique

**Affiliations:** 1 Islamic International Medical College Riphah International University Rawalpindi Pakistan Islamic International Medical College, Riphah International University, Rawalpindi, Pakistan.

Autism spectrum disorder (ASD), a complex neurodevelopmental condition characterized by impairment in social communication and repetitive behaviors affects approximately 1 in 36 children in the United States with notably higher prevalence in boys compared to girls (4:1) as per Centers for Disease Control and Prevention (CDC)’s ASD prevalence report.^1^ Psychotropics, which are conventionally used drugs to manage symptoms of ASD, often come with challenging adverse effects including metabolic syndrome, diabetes, and cardiovascular disease.^2,3^

The randomized, double-blind, and placebo-controlled clinical trial by Silva Junior et al over 12 weeks involved 60 children diagnosed with ASD, aged 5 to 11 years. The participants were divided into a treatment (31) and a control group (29). The study found that a CBD-rich cannabis extract led to statistically significant improvements in social interaction, anxiety, psychomotor agitation, and the number of meals taken/per day with only mild adverse effects reported. Additionally, a significant improvement in the “concentration” variable was observed only in children with mild ASD.^4^

The trial presents promising outcomes; however, to draw the long-term impact on developing brains and a stronger conclusion, future studies should explore the long-term effects of CBD use with larger sample sizes and broader age brackets. Among actively researched new alternative treatment options for ASD, stem cell therapy and intranasal oxytocin administration are gaining attention. Stem cell therapy particularly autologous bone marrow mononuclear cells (BMMNCs) has the potential to repair or replace damaged neural pathways associated with ASD. One such research conducted in India, exploring intrathecal administration of BMMNCs and neurorehabilitation in ASD patients observed that 94.48% and 95.27% of patients showed statistically significant improved scores on the Indian Scale for Assessment of Autism (ISAA) and Childhood Autism Rating Scale (CARS) respectively. Moreover, PET CT scans done in some of the patients before and after 6 months of intervention showed statistically significant improved brain activity in previously hypometabolized brain regions on comparative analysis and also demonstrated an increased standardized uptake value for Fluorodeoxyglucose (FDG) on PET.^5^

The study showed no significant adverse effects and one of the key findings was that a significantly better outcome of the intervention was found in patients with younger ages < 10 years and shorter duration from diagnosis < 5 years, which is why this letter advocates for early intervention with safer alternatives for ASD patients. One study supports the therapeutic potential and safety of daily administration of oxytocin, a social bonding hormone, intranasally for social cognition/function deficits and possibly repetitive behaviors in adults with ASD.^6^ However, larger sample studies of longer duration are required to fully examine these effects.

Patients undergoing treatment with cannabis-based medicinal products often face significant societal stigma,^7^ highlighting the urgent need to reduce perceived stigma at individual and community levels along with exploring alternative treatment strategies that can enhance patient acceptance. To sum up, this letter acknowledges the contribution of the authors for underscoring the critical need for ongoing research to refine and optimize treatment protocols for ASD patients and emphasizes the importance of early intervention and safer alternatives.
